# Health Predictors of Pain in Elderly—A Serbian Population-Based Study

**DOI:** 10.3390/diagnostics9020047

**Published:** 2019-04-26

**Authors:** Milena Kostadinovic, Dejan Nikolic, Dragana Cirovic, Ljubica Konstantinovic, Milica Mitrovic-Jovanovic, Natasa Radosavljevic, Mirjana Kocic, Vesna Bjegovic-Mikanovic, Milena Santric Milicevic

**Affiliations:** 1 Clinical Center of Serbia, 11000 Belgrade, Serbia; milena8250@hotmail.com; 2 Faculty of Medicine, University of Belgrade, 11000 Belgrade, Serbia; cirovicdragana@yahoo.com (D.C.); ljkonstantinovic@yahoo.com (L.K.); dr.natasa.radosavljevic@gmail.com (N.R.); 3 Physical Medicine and Rehabilitation Department, University Children’s Hospital, 11000 Belgrade, Serbia; 4 Clinic for Rehabilitation “Dr Miroslav Zotovic”, 11000 Belgrade, Serbia; 5 Department of Radiology, Clinical Center of Serbia, 11000 Belgrade, Serbia; dr_milica@yahoo.com; 6 Faculty of Medicine, University of Nis, 18000 Nis, Serbia; kocicm60@gmail.com; 7 Physical Medicine and Rehabilitation Clinic, Clinical Center Nis, 18000 Nis, Serbia; 8 Institute of Social Medicine, Faculty of Medicine, University of Belgrade, 11000 Belgrade, Serbia; bjegov@med.bg.ac.rs (V.B.-M.); milena.santric-milicevic@med.bg.ac.rs (M.S.M.)

**Keywords:** health, disease, pain, elderly

## Abstract

Objectives: The aim of our study was to evaluate the association of health factors with the presence and different degrees of pain in elderly above 65 years of life. Methods: The population-based study included 3540 individuals above 65 years of age of life from twofold stratified household sample representative for Serbia, during 2013 (the average age 73.9 ± 6.3 years; average Body Mass Index was 26.7 ± 4.4, females 56.8%, living with partner 55.5%, with primary education 55.3%, with poor wealth index 55.8% and from rural settings 46.2%). As health predictors of pain, we analyzed further health parameters: self-perceived general health, long-lasting health problems, diagnosed pulmonary disease, cardiovascular disease, musculoskeletal disease, diabetes, hyperlipidemia, hypertension and other chronic diseases. Pain domain of SF-36 version 2.0 was used for pain assessment. Results: Significant health predictors of pain were: self-perceived general health (OR 2.28), where bad perception of self-perceived general health in our study had greater risk of pain with higher degree of severity; long-lasting health problems (OR 1.60), where elderly with long-lasting health problems had almost twice the risk of moderate degree of pain, and above twice the risk for severe degree of pain; pulmonary disease (OR 1.38); musculoskeletal disease (OR 2.98) and other chronic diseases (OR 1.71). The presence of musculoskeletal disease increases the risk for pain, even more than double in severe versus mild degrees of pain. Conclusion: Bad self-perceived general health, long-lasting health problems, pulmonary, musculoskeletal diseases, cardiovascular disease and other chronic disease were significant health-related predictors of various degrees of pain in elderly.

## 1. Introduction

The prevalence of pain in the population above 60 years of age ranges between 25%–76%, and particularly in older age is associated with numerous factors including but not limited to depression, skeletal, cardiovascular and pulmonary diseases, falls, etc. [1,2]. Moreover, it was shown that a progressive increase in the prevalence of pain is in early adulthood (7%–20%), reaching a peak over the late middle age (20%–80%), while the plateau or decrease in the prevalence of pain is noticed in individuals age of 85 years and above (25%–60%) [3]. Previous studies have noticed that the rates of musculoskeletal pain are higher in older individuals [4,5]. Moreover, it is pointed out that the hallmark of osteoarthritis is persistent pain [6].

The presence of pain is associated with various degrees of disability, leading to an impaired quality of life [7]. It affects both mental and physical aspects of the quality of life [8], leading to the deconditioning, gait abnormalities, accidents and cognitive decline [9]. In a sample of elderly persons in aged care rehabilitation units only chronic physical pain, and not the intensity of pain, has an independent association with a decrease in performance [10], while in a sample of older adults attending primary health care centers, pain intensity is associated with both performance-based disability and self-reported disability [11]. The impact of pain in older individuals may limit functioning due to the fact that activity may exacerbate the pain or the elderly are afraid of reinjuries and falling [12]. In the study of Gibson and Lussier [3], above 80% of older veterans with chronic pain reported that the pain has an influence on one or more higher order physical activities, while 3% reported the influence of pain on basic activities.

It is of great importance to timely assess the proper management of pain, since it has numerous consequences namely in aged population, with dysfunctions in different degrees of functional, social and cognitive dimensions [13], deteriorating individual’s overall health, with the increase of the necessity for institutionalization, and thus increasing the health care costs. Therefore, the complexity of pain suggests an interdisciplinary approach both in diagnosis and treatment.

In Serbia, there have been a few investigations on health predictors of pain in the elderly done as case studies or series of patients. In the study of Radinovic et al. [14], it was noticed that depression and lower levels of education were predictors of pain in surgically treated hip fractured elderly in the immediate postoperative period. Further, Tripkovic et al. [15], in a cross-sectional study, showed that most frequently used medications without a doctor’s prescription were for pain relief. Population-based studies of health predictors of pain in Serbia that are conducted with representative samples are lacking. This emphasizes a need for further investigations concerning health-related predictors of pain in elderly.

We hypothesized that health factors might have an influence on the presence of pain and its degree of severity in elderly above 65 years of age. Therefore, the aim of our study was twofold: first to evaluate the association between health factors with the presence and the degree of pain in a studied group on elderly individuals and second to add to current knowledge the evidence on potential predictors of physical pain and of pain intensity at three levels (mild, moderate and severe) in a representative sample of the elderly population from Serbia. The study was an additional analysis of the third national study in Serbia “*Istraživanje zdravlja stanovništva Srbije u 2013*” [16] and was performed by the Ministry of Health of the Republic of Serbia investigating the health of Serbian inhabitants. The study largely followed the methodology and instruments of the European Health Interview Survey wave 2 (EHIS wave 2) [16,17].

## 2. Methods

### 2.1. Participants

The population-based study that was conducted in 2013 on 3540 individuals above 65 years of age from Serbia (the lowest response rate was 99.2%). Prior to inclusion into the study, eligible participants were informed about the study protocol and informed consent was obtained. The study instrument and methodology were approved by the Institutional Review Board of the Faculty of Medicine, University of Belgrade in Belgrade, Serbia (registration number 29/III-8, 13 March 2017).

### 2.2. Selection Study Criteria

For the estimation of nationally, a representative probability sample, census of individuals, households and apartments in the Republic of Serbia from 2011 was used. Stratification of the representative sample was done with regards to population data for Serbia according to the census from 2011, two variables were used for initial stratums: region and settlement type. Therefore, as main stratums of the investigated population sample, four statistical regions were identified: Vojvodina; Belgrade; Sumadija and western Serbia; and southern Serbia and eastern Serbia, that were further divided into eight stratums into cities and other areas. For the percentual representation of sample distribution on the national level, we performed two-step sampling. In the first step, we performed probability proportional sampling (in total 670 census areas). In the second step, for selected census areas we extracted households (in total 10 households and 3 additional spare households). Households were selected by the simple random sample without replacement. We gathered 6500 households in total, with 3540 (24.2% of the total population) persons with an age above 65 years of life.

The inclusion criteria for participation in the study were individuals that lived in private households and resided on the territory of the Republic of Serbia, while exclusion criteria were individuals who lived in collective households and geriatric institutions.

General characteristics of study participants are given in Table 1. They were grouped according to age into three age groups (65–74 years; 75–84 years and ≥85 years). The average age was 73.9 ± 6.3 years while the average body mass index was 26.7 ± 4.4. The study sample is composed of slightly more females (56.8%), persons living with partners (55.5%), from rural settings (46.2%), with primary education (55.3%) and with poor wealth index (55.8%) (Table 1).

### 2.3. Chronic Disease and/or Chronic Condition

According to the EHIS, chronic disease and/or chronic conditions are defined as suffering from specific disease in the past 12 months [17]. The questionnaire included presence of diagnosis for further chronic diseases or conditions: depression, cancer, urinary incontinence, kidney problems, liver cirrhosis (liver dysfunction was excluded), lower back disorder, neck disorder, arthrosis, hypertension, myocardial infarct, stroke (including chronic consequences of stroke if occurred in the past 12 months), coronary artery disease, chronic bronchitis, asthma, hyperlipidemia, diabetes (gestational diabetes excluded) and allergies [16,17]. According to interview instructions: showcard of chronic conditions and diseases was used and answers for all diseases and conditions were recorded [17]. All medical conditions were self-reported by studied individuals.

In our study, we categorized chronic diseases and conditions in seven groups: cardiovascular diseases (myocardial infarct, stroke and coronary artery disease); pulmonary disease (chronic bronchitis and asthma); diseases of musculoskeletal system (lower back disorder, neck disorder, arthrosis); diabetes; hyperlipidemia; hypertension and other chronic diseases (depression, cancer, urinary incontinence, kidney problems and liver cirrhosis).

### 2.4. Long-Lasting Health Problems

The individuals who suffered any illness or health problems over the period of at least 6 months according to the EHIS were included. The asked question was: Do you have any longstanding illness or [longstanding] health problem? and the offered answers were: yes or no [17].

### 2.5. Self-Perceived General Health

For the evaluation of self-perceived general health, we followed the recommendations of EHIS and the question was formulated “How is your health in general?” [17]. The answers were: very good; good; fair; bad; very bad [17]. For the purpose of our study, we modified the answers to three categories: good (very good and good categories), fair, and bad (bad and very bad).

### 2.6. Pain Assessment

The bodily pain dimension of the health status was assessed by version 2.0 of the SF-36 (SF-36v2), that corrected deficiencies from the original version of SF-36 bodily pain scale (SF-36v1 BPS) [18]. SF-36 BPS is a two item scale with six outcomes (none; very mild; mild; moderate; severe; very severe) where the question asked referred to the pain that was in the past 4 weeks [19]. For the purpose of our study, we modified the outcome to four categories (none; mild; moderate and severe), where mild category included: very mild and mild categories, and severe category: severe and very severe categories.

### 2.7. Statistical Analysis

Categorical variables are presented as absolute and relative frequency, and for testing the statistical significance between them we used a chi-squared test. For identification of factors that are independent predictors of pain intensity, we used univariate logistic regression and multivariate logistic regression that included variables from univariate logistic regression with *p* < 0.05. For quantification of the strength of association of significant predictors and pain intensity, we used Odds ratio (OR) with 95% confidence interval (CI). The four models were extracted: Model 1—between the group with no pain and mild pain; Model 2—between the group with no pain and moderate pain; Model 3—between the group with no pain and severe pain; and Model 4—between the group with no pain and with pain. The statistical significance was set at *p* < 0.05.

The number of evaluated chronic diseases and conditions was reduced and grouped into: other chronic conditions; musculoskeletal; cardiovascular; pulmonary; and metabolic diseases, by the application of exploratory factorial analysis with the extraction of factors by analysis of main components and Varimax orthogonal rotations of factors. Implementation of factorial analysis was justified by the Bartlett’s test of sphericity (*p* < 0.001), and the sample is adequate since the Kaiser-Meyer-Olkin test has *p* = 0.779. The criteria for the number of factors is the value of latent root (eigenvalue) >1, and factorial burden of variables within factors >0.4 (Table 2 and Table 3).

## 3. Results

### 3.1. Data Grouping

Initial eigenvalues as well as values after rotation are presented in Table 2, while in Table 3 we presented the factorial matrix after orthogonal rotation. Therefore, further chronic diseases and conditions and groups were evaluated: cardiovascular diseases (myocardial infarct, stroke and coronary artery disease); pulmonary disease (chronic bronchitis and asthma); diseases of musculoskeletal system (lower back disorder, neck disorder, arthrosis); diabetes; hyperlipidemia; hypertension and other chronic diseases (depression, cancer, urinary incontinence, kidney problems and liver cirrhosis).

### 3.2. Health Parameters

The pain of various degrees was present in 66.4% of elderly (19.4% of mild, 24.5% of moderate and 22.5% of severe degree). In Table 4, frequencies of health variables in relation to the pain intensity are presented.

There were 789 (22.3%) individuals with good, 1334 (37.7%) with fair and 1416 (40.0%) with bad self-perceived general health. Long-lasting health problems were noticed in 2679 (75.9%) participants. Pulmonary disease was reported in 417 (11.8%), cardiovascular in 2568 (72.6%), musculoskeletal in 1629 (46.1%), diabetes in 639 (18.2%), hyperlipidemia in 751 (22.0%), hypertension in 2348 (66.6%) and other chronic diseases in 1182 (33.4%) individuals.

For self-perceived general health, there were significant differences in frequencies of different categories of such variable regarding all pain categories separately (*p* < 0.001), with the highest proportion of fair category in the groups with no pain and mild degree of pain, and bad category in the groups with moderate and severe degrees of pain. Further, significant differences in frequencies of patients in defined categories of self-perceived general health were noticed regarding the presence and different degrees of pain (good and bad categories with *p* < 0.001 and fair category with *p* < 0.01), with the highest frequency of no pain in good and fair categories, and severe pain in bad category.

There was a significant difference in frequencies of patients with long-lasting health problems regarding the presence and different degrees of pain (*p* < 0.001), with the highest frequency for patients with a severe degree of pain.

Significant differences in frequencies of different categories of chronic diseases and/or conditions were present for all pain categories separately (*p* < 0.001), with the highest frequency of cardiovascular diseases, followed by hypertension and musculoskeletal diseases. In addition, significant differences in frequencies of patients in defined categories of chronic diseases and/or conditions were noticed regarding the presence and different degrees of pain (for diabetes and hyperlipidemia *p* < 0.05; pulmonary and cardiovascular diseases *p* < 0.01; and musculoskeletal diseases, hypertension and other chronic diseases *p* < 0.001), with the highest frequency of severe degree of pain for pulmonary diseases, musculoskeletal diseases, hyperlipidemia and other chronic diseases, and no pain for cardiovascular diseases, diabetes and hypertension.

In Table 5, health factors significantly associated with the presence and degree of pain by univariate and multivariate logistic regression analysis are presented. After applying variables that were significantly associated with pain of different intensities in the evaluated models, from univariate into multivariate logistic regression analysis, self-perceived general health, musculoskeletal disease and other chronic diseases were significantly associated with all models of pain intensity, while long-lasting health problems were significantly associated with Models 2, 3 and 4, pulmonary disease was significantly associated with Models 1, 3 and 4, cardiovascular disease was significantly associated only with Model 1 (Table 5). Diabetes, hyperlipidemia and hypertension were not significantly associated with evaluated models.

## 4. Discussion

In our study, we have demonstrated that further health parameters: bad self-perceived general health, long-lasting health problems, pulmonary, cardiovascular, and musculoskeletal diseases, and other chronic diseases were significant predictors of pain in elderly. Moreover, some conditions of above mentioned diseases could be considered as symptom of pain, thus multidisciplinary and individualized approach to the patient’s condition is advised.

The bad perception of self-perceived general health in our study was significantly correlated with pain in our study, where the greatest positive correlation was with pain with a higher degree of severity. This dimension tends to be a good predictor of objective health in the aged population, including the number of comorbidities and functional disability degree [20]. Further, we noticed that those with good perception of self-perceived general health (22.3% of respondents) most frequently had no pain (around one third of studied sample), those with fair perception most frequently had mild degree (close to half of studied sample), while those with bad perception most frequently had severe degree of pain (just below two thirds of studied sample). These findings could be explained by the fact that aged individuals usually have comorbidities that influence one’s perception of general health. Moreover, elderly tend to have more than one comorbidity and thus poorer perception of general health. In line with such observation, Hermsen et al. found that more than two chronic diseases were found in almost half of the studied population of elderly [21]. The literature also showed that health standards probably depend very much on gender [22], age [23,24] and experience of coping with health problems [24]. In addition, when assessing their health and pain, participants also might have included aspects that go beyond the usually analyzed factors such as being illness-prone, have learned to live with its limitations, social comparison, positive attitude towards the condition, etc. [25]. Other characteristics of many of our study participants need to be considered as well as potential confounding factors for pain assessment. Such confounding factors include a low level of education, emotional suffering of single elderly, a low wealth index, and rural residence because they might negatively affect the control over pain in two ways; directly [26,27,28,29], or indirectly through inequality in access to quality health care and medicines. In both cases, these factors might contribute to under or over-estimation of pain reporting in this cross-sectional study.

Long-lasting health problems in our study were significantly associated with pain, in particular with moderate and severe degrees of pain. Elderly with long-lasting health problems had almost twice the risk of a moderate degree of pain, and above twice the risk for a severe degree of pain. The aged population has numerous chronic diseases and conditions including: peripheral neuropathies, advanced heart disease, advanced chronic obstructive pulmonary disease, osteoarthritis, irritable or inflammatory bowel disease and others [30]. They, thus, might have an influence not just on the onset of the pain, but the degree of pain severity as well. Such influence could be cumulative in a temporal sense (duration of health problem) and dimensional point (several comorbidities). In the study of Ciesielczyk et al., it was noticed that pain hypersensitivity is associated with gastrointestinal disorders [31]. Moreover, other factors should be taken into consideration in elderly as well, among them are maladaptive coping strategies and improper social support that could have an influence on pain severity degree among other chronic diseases and conditions [25]. This clearly points out how complex and multidimensional the influence of certain conditions and diseases are in the aged population in relation to the degree pain severity.

Among the evaluated diseases, musculoskeletal disease and other chronic diseases are considered significantly associated with pain in elderly. Previously it was stated that in the aged population with chronic musculoskeletal conditions, pain is the primary complaint, with most individuals been presented with a non-specific type of the pain [32]. Since the aged population is prone to falls, complex regional pain syndrome is one of the commonly encountered conditions after fractures, particularly distal radial fractures [33]. Further, we demonstrated that for musculoskeletal disease the risk for severe versus mild degrees is greater more than double. The same trend was noticed for other chronic diseases, with less than double between severe versus mild degrees. It is known that patients with cancer commonly experience pain as a symptom, and it is worth mentioning that the incidence of cancer increases with age [34]. Moreover, these patients might present with acute or persistent pain. Furthermore, metastases in elderly are an important etiology of pain, but such a symptom could be caused indirectly by treatment [35,36]. Among other chronic diseases, depression should be mentioned as well, since in the aged population, such condition is strongly associated with pain severity [36,37].

There were several limitations to this study. The medical conditions were reported from the questionnaire by a self-reporting method of the studied individuals. Even though the sampling models for an adequate study sample were done, the cross-sectional design of our study did not allow assuming the cause-effect relations among pleiotropic causes of pain and number of tested variables from our study might be taken into consideration in future studies. The study findings may encompass bias related to memory recall of respondents or the anticipation that the questionnaire used in our study describes good enough the self-perception of the pain in elderly participants. Also, the pain category was analyzed from the pain domain of SF-36 version 2. The study results cannot be generalized to the elderly in other settings such as nursing homes, shelters etc., nor to others, apart from the variables described.

Since the pain prevalence is high among the elderly, apart from therapy for medical conditions and pharmacotherapy, the pain management should consider the effectiveness, the presence of adverse effects and suitability of nonpharmacologic approaches such as psychosocial interventions, cognitive behavioral therapy, complementary and alternative modes of therapy, and osteopathic manipulative treatment [38,39,40,41,42,43,44].

In conclusion, bad self-perceived general health, long-lasting health problems, pulmonary, musculoskeletal diseases and other chronic disease are significant health-related predictors of pain. The cardiovascular disease might be considered as a health-related predictor of a mild degree of pain in the elderly. Our findings might point to the fact that studied predictors could have an influence on the different degrees not solely for pain onset, but on its severity degree in the aged population.

## Figures and Tables

**Table 1:** Patient characteristics.

Parameters		65–74 Years (*n* = 1955)	75–84 Years (*n* = 1385)	≥85 Years (*n* = 200)	Total (*n* = 3540)
Age, years *(MV ± SD)		69.2 ± 3.0	78.6 ± 2.7	87.4 ± 2.6	73.9 ± 6.3
Height, cm *(MV ± SD)		168.2 ± 9.3	166.7 ± 9.6	165.2 ± 9.8	167.5 ± 9.5
Weight, kg *(MV ± SD)		77.0 ± 13.9	72.3 ± 13.5	65.3 ± 13.7	74.6 ± 14.0
Body Mass Index, kg/m^2^ *(MV ± SD)		27.3 ± 4.5	26.1 ± 4.2	24.1 ± 3.5	26.7 ± 4.4
Gender	Females, *n* (%)	1080 (55.2)	796 (57.5)	136 (68.0)	2012 (56.8)
	Males, *n* (%)	875 (44.8)	589 (42.5)	64 (32.0)	1528 (43.2)
**Education level, *n* (%)**					
Primary		960 (49.1)	853 (61.6)	143 (71.5)	1956 (55.3)
High school		708 (36.2)	364 (26.3)	32 (16.0)	1104 (31.2)
College/University		287 (14.7)	168 (12.1)	25 (12.5)	480 (13.6)
**Marital status, *n* (%)**					
Single		25 (1.3)	16 (1.2)	2 (1.0)	43 (1.2)
Married/Living with a partner		1293 (66.1)	629 (45.4)	43 (21.5)	1965 (55.5)
Widow/Widower		560 (28.6)	713 (51.5)	152 (76.0)	1425 (40.3)
Divorced		77 (3.9)	27 (1.9)	3 (1.5)	107 (3.0)
**Wealth Index, *n* (%)**					
Poor		1021 (52.2)	835 (60.3)	119 (59.5)	1975 (55.8)
Middle		374 (19.1)	226 (16.3)	31 (15.5)	631 (17.8)
High		560 (28.6)	324 (23.4)	50 (25.0)	934 (26.4)
**Residence, *n* (%)**					
City		1043 (53.4)	762 (55.0)	98 (49.0)	1903 (53.8)
Rural		912 (46.6)	623 (45.0)	102 (51.0)	1637 (46.2)

* Mean Values (MV); Standard Deviation (SD).

**Table 2:** Eigenvalues and variances within factors.

Initial Eigenvalues			Values after Rotation		
Total	% of Variance	Cumulative %	Total	% of Variance	Cumulative %
3.561	20.948	20.948	1.924	11.317	11.317
1.528	8.988	29.936	1.835	10.793	22.110
1.328	7.810	37.746	1.694	9.965	32.075
1.164	6.849	44.596	1.604	9.436	41.511
1.007	5.925	50.521	1.532	9.010	50.521

**Table 3:** Factorial matrix after orthogonal rotation.

Variables	Factors and Factorial Burden of Selected Variables within Factors				
	Other Chronic Disease	Musculoskeletal Diseases	Cardiovascular Disease	Pulmonary Diseases	Metabolic Diseases
Depression	0.667	-	-	-	-
Cancer	0.603	-	-	-	-
Urinary incontinence	0.590	-	-	-	-
Kidney problems	0.573	-	-	-	-
Liver cirrhosis	0.417	-	-	-	-
Lower back disorder	-	0.858	-	-	-
Neck disorder	-	0.835	-	-	-
Arthrosis	-	0.565	-	-	-
Hypertension	-	-	0.726	-	-
Myocardial infarct	-	-	0.677	-	-
Stroke	-	-	0.670	-	-
Coronary artery disease	-	-	0.423	-	-
Chronic bronchitis	-	-	-	0.877	-
Asthma	-	-	-	0.870	-
Hyperlipidemia	-	-	-	-	0.685
Diabetes	-	-	-	-	0.464
Allergy	-	-	-	-	-

**Table 4:** Frequencies of health variables in relation to the pain intensity.

Variables	Presence of Pain During the Last 4 Weeks					
	No Pain *N* (%)	Mild Pain *N* (%)	Moderate Pain *N* (%)	Severe Pain *N* (%)	Total *N* (%)	*p*
**Self-perceived general health**						
Good	481 (40.6)	164 (23.9)	100 (11.5)	44 (5.5)	789 (22.3)	<0.001
Fair	507 (42.7)	334 (48.6)	331 (38.2)	162 (20.3)	1334 (37.7)	<0.01
Bad	200 (16.8)	189 (27.5)	435 (50.2)	592 (74.2)	1416 (40.0)	<0.001
*p*	<0.001	<0.001	<0.001	<0.001	<0.001	
**Long-lasting health problems**	706 (59.5)	497 (72.8)	737 (85.6)	739 (92.7)	2679 (75.9)	<0.001
**Chronic disease and/or conditions**						
Pulmonary disease	77 (6.5)	78 (11.4)	129 (15.0)	133 (16.7)	417 (11.8)	<0.01
Cardiovascular disease	731 (61.8)	495 (72.1)	673 (77.8)	669 (83.8)	2568 (72.6)	<0.01
Musculoskeletal disease	276 (23.3)	281 (40.9)	486 (56.3)	586 (73.5)	1629 (46.1)	<0.001
Diabetes	192 (16.2)	108 (15.8)	158 (18.4)	181 (22.9)	639 (18.2)	<0.05
Hyperlipidemia	199 (17.2)	137 (20.4)	203 (24.5)	212 (27.8)	751 (22.0)	<0.05
Hypertension	676 (57.1)	453 (66.3)	614 (71.2)	605 (76.0)	2348 (66.6)	<0.001
Other chronic diseases	224 (18.8)	211 (30.7)	319 (36.9)	428 (53.7)	1182 (33.4)	<0.001
*p*	<0.001	<0.001	<0.001	<0.001	<0.001	

**Table 5:** Health factors significantly associated with the presence and degree of pain by univariate and multivariate logistic regression analysis. OR: odds ratio; CI: confidence interval.

Variables	Model 1 between Group with No Pain and Mild Pain OR (95% CI)	Model 2 between Group with No Pain and Moderate Pain OR (95% CI)	Model 3 between Group with No Pain and Severe Pain OR (95% CI)	Model 4 between Group with No Pain and with Pain OR (95% CI)
**Univariate analysis**				
**Self-perceived general health**	1.68 ** (1.47–1.92)	3.25 *** (2.84–3.71)	6.71 *** (5.66–7.97)	3.08 *** (2.78–3.41)
**Long-lasting health problems**	1.82 *** (1.48–2.23)	4.05 *** (3.24–5.06)	8.69 *** (6.49–11.62)	3.65 *** (3.11–4.29)
**Pulmonary diseases**	1.86 *** (1.33–2.58)	2.55 *** (1.89–3.43)	2.90 *** (2.154–3.90)	2.45 *** (1.89–3.17)
**Cardiovascular disease**	1.62 *** (1.32–1.98)	2.20 *** (1.80–2.68)	3.25 *** (2.61–4.06)	2.24 *** (1.93–2.61)
**Musculoskeletal disease**	2.28 *** (1.87–2.80)	4.24 *** (3.51–5.13)	9.10 *** (7.40–11.19)	4.49 *** (3.83–5.25)
**Diabetes**	0.97 (0.75–1.25)	1.16 (0.92–1.46)	1.53 *** (1.22–1.92)	1.22 * (1.01–1.47)
**Hyperlipidemia**	1.24 (0.97–1.58)	1.57 *** (1.26–1.95)	1.87 *** (1.50–2.32)	1.56 *** (1.30–1.87)
**Other chronic diseases**	1.91 *** (1.54–2.37)	2.52 *** (2.06–3.08)	4.98 *** (4.07–6.09)	2.97 *** (2.51–3.51)
**Hypertension**	1.48 *** (1.21–1.80)	1.86 *** (1.54–2.24)	2.38 *** (1.95–2.90)	1.87 *** (1.62–2.17)
**Multivariate analysis**				
**Self-perceived general health**	1.43 *** (1.24–1.65)	2.54 *** (2.18–2.96)	4.09 *** (3.36–4.96)	2.28 *** (2.03–2.57)
**Long-lasting health problems**	-	1.85 *** (1.43–2.40)	2.49 *** (1.73–3.57)	1.60 *** (1.32–1.94)
**Pulmonary disease**	1.50 * (1.06–2.13)	-	1.56 * (1.05–2.33)	1.38 * (1.03–1.85)
**Cardiovascular disease**	1.27 * (1.03–1.59)	-	-	-
**Musculoskeletal disease**	1.83 *** (1.48–2.26)	3.19 *** (2.58–3.94)	5.28 *** (4.12–6.77)	2.98 *** (2.51–3.55)
**Diabetes**	-	-	-	-
**Hyperlipidemia**	-	-	-	-
**Other chronic diseases**	1.49 ** (1.19–1.88)	1.61 *** (1.28–2.30)	2.32 *** (1.79–3.00)	1.71 *** (1.40–2.07)
**Hypertension**	-	-	-	-

* *p* < 0.05; ** *p* < 0.01; *** *p* < 0.001.
